# Prediction of the Non-Isothermal Austenitization Kinetics of Fe-C-Cr Low Alloy Steels with Lamellar Pearlite Microstructure

**DOI:** 10.3390/ma15062131

**Published:** 2022-03-14

**Authors:** Zhiqiang Li, Shengyang Zhang, Yang He, Guangjie Xiong, Yude Liu, Fuyong Su

**Affiliations:** 1 School of Artificial Intelligence, Beijing Technology and Business University, Beijing 100048, China; zhql041989@126.com (Z.L.); guangjiexiong@126.com (G.X.); yudeliu1992@126.com (Y.L.); 2 Institute for Advanced Materials and Technology, University of Science and Technology Beijing, Beijing 100083, China; 3 School of Energy and Environmental Engineering, University of Science and Technology Beijing, Beijing 100083, China; sfyong@ustb.edu.cn; 4 Beijing Key Laboratory of Energy Saving and Emission Reduction for Metallurgical Industry, University of Science and Technology Beijing, Beijing 100083, China

**Keywords:** austenitization, heating rate, coupling diffusion, pearlite lamellae orientation, 3D cellular automata simulation

## Abstract

The austenitization of low alloy steels during rapid heating processes was involved in many kinds of advanced heat treatment technologies. Most of the previous research on the austenitization kinetics was focused on the spherical pearlite microstructures, which were different from the lamellar pearlite microstructures. In the present research, to predict the non-isothermal austenitization process of an Fe-C-Cr steel with lamellar pearlite, a novel 3-dimensional (3D) cellular automata model, which considered the influences of the coupling diffusion of Cr and C, and the interfacial diffusion between pearlite lamellae and the pearlite lamellar orientation, was established based on the thermodynamic equilibrium data obtained from the Thermo-Calc software and the simulation results of the DICTRA module. To clarify the influences of the heating rate on the austenitization kinetics and validate the simulation results, the austenitization processes of a Fe-1C-1.41Cr steel for different heating rates were studied with a series of dilatometric experiments. The good agreements between the cellular automata simulation results and the experimental results showed that the newly proposed cellular automata model is reasonable. The experimental results show an obvious change of the transition activity energies from the low to high heating rates. The transition from partitioning local equilibrium (PLE) to non-partitioning local equilibrium (NPLE) mechanisms was proved with DICTRA simulations. Basing on the simulation results, the influences of the pearlite lamellae orientation on the austenitization kinetics and the topological aspects of austenite grains were evaluated. In addition, the topological aspects of the rapidly austenitized grains were also compared to the normal grains.

## 1. Introduction

Many advanced heat treatment technologies for steels, such as laser quenching [1,2], welding [3], coating [4] and rapid transformation annealing [5], contain fast-heating processes. The fast-heating technologies not only promote energy conservation and production efficiency improvement, but also lead to different composition and microstructure distributions [6,7,8] and mechanical properties. During the fast-heating processes, the pearlite to austenite transformations of the low alloy steels were carried out in wide temperature ranges. Therefore, the non-isothermal austenitization processes were paid more and more attention.

Previous research showed that the improving of the austenitization temperature obviously changed the pearlite to austenite transformation kinetics. This was related to the different coupling diffusion mechanisms of the substitutional elements and C at different temperatures. Miyamoto et al. [9] studied the austenitization of low alloy steels containing Mn and Cr from a pearlite structure that consisted of a spheroidized cementite and ferrite matrix. Their STEM/EDS analyses revealed that the austenite/cementite interface migrated without Mn partitioning; however, the migration of the austenite/cementite interface was accompanied by Cr partitioning and was very inhibited. The local equilibrium model simulations showed that the partitioning local equilibrium (PLE) and non-partitioning local equilibrium (NPLE) of substitutional alloys could both occur during the migration of *γ*/*α* and *γ*/*θ* interfaces [10,11,12,13,14,15]. Xia et al. [10] proposed that the PLE occurred at low overheating and the NPLE occurred at high overheating. There existed a partition to no-partition transition temperature (PNTT) between the two transition modes. In non-isothermal processes, the austenitization temperature regions of steels obviously increased with the heating rate [16]; thus, the fast-heating rate would lead to high overheating. When the austenitization temperature exceeded the PNTT, the non-partition transformation occurred, and the transformation rate was improved. Therefore, to predict the non-isothermal austenitization kinetics, the partition to non-partition transition mechanism during the heating process should be considered.

To our knowledge, most of the studies on the influences of substitutional alloys’ partition during reverse transformation were based on pearlite with spheroidized cementite, but the research on lamellar pearlite was rare. Our previous research showed that increasing the heating rate of Fe-C-Cr bearing steel with an initial microstructure of lamellar pearlite also abnormally improved the austenitization rate [17]. For the lamellar pearlite, the lamellar spacing and the interfacial diffusion of the solutes should influence the pearlite to austenite transformation rate [18]. As the lamellae of pearlite have certain orientations, the relationship between the austenite growth direction and lamellar orientation also influences the transformation rate. The scale of the pearlite colony is much larger than the lamellar spacing; therefore, a model, which considers both the solute coupling diffusion between pearlite lamellas and the dissolution of a large number of pearlite colonies to predict the non-isothermal austenitization processes of low alloy steels is difficult and still lacking.

As the most widely used bearing steel in the world, GCr15 bearing steel has been researched for about 100 years. Its fatigue life increased in multiples with the improvement of technologies. In recent years, GCr15 bearing steel is still subject to many researchers [19,20,21,22]. For the popularity and simple compositions of GCr15 bearing steel, this steel was used as the model material in the present research. To predict the non-isothermal austenitization of this steel, a 3D cellular automata model, which considers the influences of coupling diffusion of Cr and C, the interfacial diffusion between pearlite lamellae and the pearlite lamellar orientations, was established basing on the thermodynamic equilibrium data obtained from the Thermo-Calc software and the simulation results of the DICTRA module. To clarify the influence of heating rate on the austenitization kinetics and provide some validation data for the simulation results, the austenitization experiments for different heating rates were carried out firstly.

## 2. Materials and Methods

### 2.1. Experimental

In order to obtain the influences of heating rate on the austenitization kinetics and provide validation data for the simulation results, the experimental research for different heating rates was carried out. The commercial GCr15 bearing steel (the corresponding designations are AISI/SAE 52100 and DIN 100Cr6) was utilized as the research object. The composition of the material was 1.0 C, 1.42 Cr, 0.35 Mn, 0.25 Si, 0.025 P, 0.025 S, 0.3 Ni, 0.25 Cu and 0.10 Mo wt.%. To obtain a uniform pearlitic microstructure, the samples cut from the columnar zone of a continuous casting billet were held at 1150 °C for 2 h then air cooled to room temperature. This heat treatment experiment was carried out in the School of Energy and Environmental Engineering of the University of Science and Technology Beijing. The samples were then cut to the size of 4 mm in diameter and 10 mm in length. During the experimental processes, the samples were heated under vacuum protection to be austenitized, then quenched with nitrogen after the objective temperature was achieved. A DIL 805A-type dilatometer was used to measure the length changes of the samples during the uniform heating processes. The temperatures of the samples were also measured with a Pt Rh10-Pt thermocouple. The heating rates of the samples were 0.13, 0.27, 0.53, 1.07, 3, 30, 60 and 120 °C·s^−1^. All the samples were heated to temperatures over 900 °C to ensure the complete dissolution of ferrite. The dilatometric experiments were carried out in the Institute of Engineering Technology of the University of Science and Technology Beijing. As the pearlite consisted of cementite and ferrite, the deviations of the length–temperature curves of the samples from a straight line during the austenitization processes were related to differences of the lattice constants between the pearlite and austenite. To derive the transformed austenite fraction from the length–temperature curves of the samples, a previous proposed model considering the lattice constants of the three phases was used.

### 2.2. Modeling

#### 2.2.1. DICTRA Model

The DICTRA module of the commercial Thermo-Calc software was used to simulate the dissolution of the ferrite and cementite plates to austenite. The infinite plate diffusion model and the TCFE6 (thermodynamic) and MOB2 (dynamic) databases were adopted. The thicknesses of the ferrite and cementite plates were 0.102 and 0.018 μm. The initial austenite plate with a thickness of 0.001 μm was located between the ferrite and cementite plates. To simplify the calculation, the model only considered the main compositions of the steel: Fe, C and Cr. According to the research of Razik et al. [23], the partition coefficient for Cr in the pearlite was about 1.1 when the pearlite was obtained with a normal cooling rate. Thus, the initial Cr concentrations of the ferrite and cementite were calculated to be 1.41 wt.% and 1.45 wt.%, respectively. As the C concentration of the cementite was fixed to ~6.74 wt.%, the C concentration of the ferrite was calculated to be 0.0069 wt.%. The initial concentrations of C and Cr of the austenite were 1 wt.% and 1.42 wt.%, respectively. 

#### 2.2.2. Cellular Automata Model

1. Nucleation

The nucleation of austenite can be divided into two patterns: continuous nucleation and instantaneous nucleation. For the continuous nucleation, the austenite nuclei produce in throughout the austenitization process; for the instantaneous nucleation, the nucleation sites are instantaneously depleted at the beginning of the austenitization process. The research of Dernfeld [24] confirmed that the two nucleation patterns were mainly decided by the concentration of C. The research of Speiche [25] showed instantaneous nucleation of austenite from the pearlitic microstructure with 0.96 wt.% C, which was very close to the present steel with 1 wt.% C. Thus, the instantaneous nucleation was adopted in the present cellular automata model. As the austenite nuclei can generate at angles and boundaries of pearlite colony, interfaces of pearlite lamella and boundaries of secondary cementite, it is difficult to calculate the nucleation density with a parametric model. Thus, the nucleation density of the present steel was obtained to be 1.69 × 10^15^ m^−3^ by directly measuring the austenite grain density of a just austenitized microstructure.

2. Austenite/pearlite interface moving velocity

**Figure 1:**
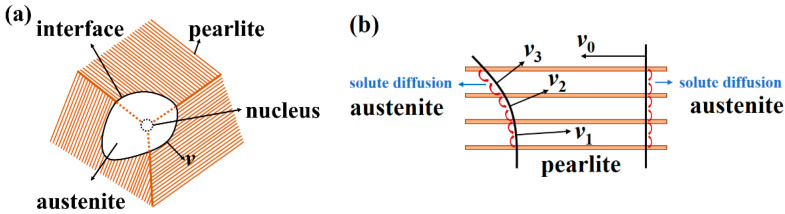
Schematic diagram for the austenitization of lamellar pearlite. (**a**) Growth of an austenite grain in pearlite matrix; (**b**) influence of pearlite lamellae orientation on the γ/P interface moving velocity.

The rate of pearlite to austenite transformation depends on the austenite nuclei density and moving velocity of the austenite–pearlite interface. As shown in Figure 1a, after the austenite nucleus was generated, the austenite/pearlite interface would move to the surrounding pearlite plates. As shown in Figure 1b, the moving velocity of the interface was defined by the diffusion of solute, from cementite to ferrite, through the formed austenite volume [26] and the austenite/ferrite (*γ/α*) interface [27]. If the *γ/α* interface was perpendicular to the pearlite lamellas, the interface would move with the fastest velocity for the shortest solute diffusion distance. However, if the interface was not perpendicular to the pearlite lamellas, the interface would move with a slower velocity for the increasing of the solute diffusion distance. According to the above analysis, the transformation of pearlite to austenite was a complicated process containing the interactive mechanisms between nucleation location and interface moving velocity. 

As shown in Figure 1b, the following formula was used to calculate the interface moving velocity:

(1) $$v=v_{0}\cos\phi ,$$

(2) $$\cos\phi =\frac{\left|\vec{n_{1}}\cdot \vec{n_{2}}\right|}{\left|\vec{n_{1}}\right|\cdot \left|\vec{n_{2}}\right|},$$

where *v*_0_ was the interface moving velocity parallel to the pearlite plates; $\vec{n_{1}}$ and $\vec{n_{2}}$ were the normal vector of pearlite plates and moving direction vector of austenite/pearlite (*γ*/*P*) interface; *ϕ* was the included angle between the two vectors.

**Figure 2:**
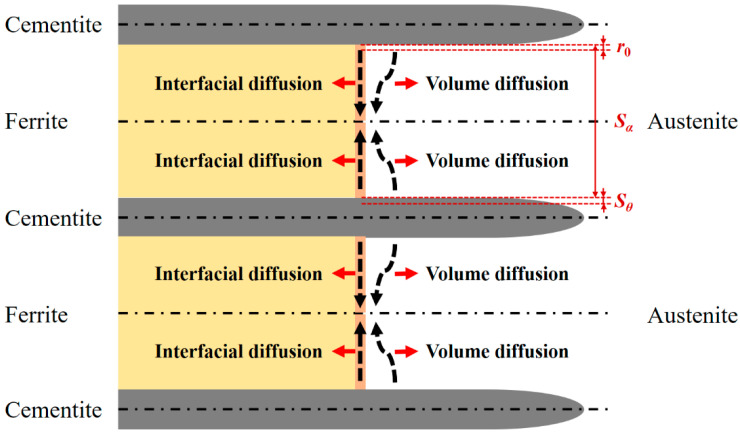
Schematic diagram for the volume and interfacial diffusions between the ferrite and cementite lamellae.

During the non-isothermal process, the interfacial moving velocity is related to the temperature and the diffusion of solutes between the phases. Figure 2 shows the diffusion of the solutes between cementite and ferrite. As shown in the figure, the solutes diffused through the volume of austenite [26] and the interface between ferrite and austenite [27]. The two diffusion paths both had significant impacts on the austenitization process. As the solute diffusion coefficients in cementite and ferrite were much smaller than in austenite, the solute diffusions through cementite and ferrite were neglected. The flux of solute diffusing through the volume of austenite, *J_V_*, was calculated with the following equation:

(3) $$J_{V}=\frac{A_{\alpha }}{\frac{S_{\alpha }}{2}-r_{0}}D^{V}\left[\ln\left(\frac{S_{\alpha }}{2}\right)-\ln r_{0}\right]\left(c^{\gamma \theta }-c^{\gamma \alpha }\right),$$

where *A_α_* was the interfacial area which the solute diffused into. For a unit depth, *A_α_* equaled the thickness of the ferrite lamella; *S_α_*. *r*_0_ was the minimum distance from cementite to ferrite, which was considered a few cell parameters in thickness, 10^−8^ m; *D^V^* was the diffusion coefficient of the solute in austenite; *c_γθ_* and *c_γα_* were the solute concentration in austenite at the austenite/cementite (*γ*/*θ*) interface and *γ*/*α* interface, respectively. The flux of solute diffusing through the *γ*/*α* interface, *J_I_*, could be calculated with the following formula:

(4) $$J_{I}=\frac{12k_{1}D^{I}\delta (c^{\gamma \theta }-c^{\gamma \alpha })}{S_{\alpha }},$$

where *k*_1_ was the boundary segregation coefficient of the interface; *D^I^* was the solute diffusion coefficient in the interface; *δ* was the thickness of the interface, which was always evaluated to be 2.5 Å. *k*_1_*D^I^δ* was usually set as one parameter and can be evaluated from the experimental data. 

When the temperature is under the PNTT, the partition of the substitutional alloying element is necessary. Thus the austenitization process was controlled by the couple diffusion of C and Cr, and the following equations could be obtained according to the solute flux balance:

(5) $$v_{0}S_{\alpha }(c_{C}^{\gamma \alpha }-c_{C}^{\alpha \gamma })=J_{V}^{C}+J_{I}^{C}=(c_{C}^{\gamma \theta }-c_{C}^{\gamma \alpha })\left(\frac{A_{\alpha }}{\frac{S_{\alpha }}{2}-r_{0}}D_{C}^{V}\left(\ln\left(\frac{S_{\alpha }}{2}\right)-\ln r_{0}\right)+\frac{12kD_{C}^{I}\delta }{S_{\alpha }}\right),$$

(6) $$v_{0}S_{\alpha }(c_{Cr}^{\gamma \alpha }-c_{Cr}^{\alpha \gamma })=J_{V}^{Cr}+J_{I}^{Cr}=(c_{Cr}^{\gamma \theta }-c_{Cr}^{\gamma \alpha })\left(\frac{A_{\alpha }}{\frac{S_{\alpha }}{2}-r_{0}}D_{Cr}^{V}\left(\ln\left(\frac{S_{\alpha }}{2}\right)-\ln r_{0}\right)+\frac{12kD_{Cr}^{I}\delta }{S_{\alpha }}\right),$$

(7) $$v_{0}S_{\theta }(c_{C}^{\theta \gamma }-c_{C}^{\gamma \theta })=J_{V}^{C}+J_{I}^{C}=(c_{C}^{\gamma \theta }-c_{C}^{\gamma \alpha })\left(\frac{A_{\alpha }}{\frac{S_{\alpha }}{2}-r_{0}}D_{C}^{V}\left(\ln\left(\frac{S_{\alpha }}{2}\right)-\ln r_{0}\right)+\frac{12kD_{C}^{I}\delta }{S_{\alpha }}\right),$$

(8) $$v_{0}S_{\theta }(c_{Cr}^{\theta \gamma }-c_{Cr}^{\gamma \theta })=J_{V}^{Cr}+J_{I}^{Cr}=(c_{Cr}^{\gamma \theta }-c_{Cr}^{\gamma \alpha })\left(\frac{A_{\alpha }}{\frac{S_{\alpha }}{2}-r_{0}}D_{Cr}^{V}\left(\ln\left(\frac{S_{\alpha }}{2}\right)-\ln r_{0}\right)+\frac{12kD_{Cr}^{I}\delta }{S_{\alpha }}\right),$$

where *S_θ_* represented the thickness of the dissolved cementite during the ferrite to austenite transformation. As the cementite could not be completely dissolved during the ferrite to austenite transformation process, the value of *S_θ_* was unknown. *J_V_^C^* and *J_I_^C^* were the flux of C diffusing through the volume of austenite and the *γ*/*α* interface, respectively. *J_V_^Cr^* and *J_I_^Cr^* were the flux of Cr diffusing through the volume of austenite and the *γ*/*α* interface, respectively. The expression *c_E_^pq^* indicated the concentration of solute *E* in phase *p* at the interface between phase *p* and *q*. Where *E* expressed C or Cr, *p* and *q* expressed the ferrite (*α*), cementite (*θ*) or austenite (*γ*). The expressions *D_E_^V^* and *D_E_^I^* respectively indicated the volume and interfacial diffusion coefficients of solute *E*. The values of *D_C_^V^* and *D_Cr_^V^* could be calculated with the following formulas [28]:

(9) $$D_{C}^{V}=D_{C0}^{V}\exp\left(-\frac{Q_{C}^{V}}{RT}\right),$$

(10) $$D_{Cr}^{V}=D_{Cr0}^{V}\exp\left(-\frac{Q_{Cr}^{V}}{RT}\right),$$

where *D_C_*_0_*^V^* and *D_Cr_*_0_*^V^* were frequency factors, equal to 1.75 × 10^−5^ and 1.43 × 10^−4^ m^2^·s^−1^ for C and Cr, respectively. *Q_C_^V^* and *Q_Cr_^V^* were diffusion activation energies, equal to 143,320 and 284,470 J·mol^−1^ for C and Cr, respectively. *R* was the universal gas constant, equal to 8.314 J·mol^−1^·K^−1^. *T* was the temperature, and had the unit of K. The values of the expressions *kD_C_^I^δ* and *kD_Cr_^I^δ* could be calculated with the following equations [29,30]:

(11) $$k_{1}D_{C}^{I}\delta =D_{C0}^{I}\exp\left(-\frac{Q_{C}^{I}}{RT}\right),$$

(12) $$k_{1}D_{Cr}^{I}\delta =D_{Cr0}^{I}\exp\left(-\frac{Q_{Cr}^{I}}{RT}\right),$$

where *D_C_*_0_*^I^* and *D_Cr_*_0_*^I^* were pre-exponential factors, equal to 2.13 × 10^−14^ and 5.14 × 10^−14^ m^3^ s^−1^, respectively; *Q_C_^I^* and *Q_Cr_^I^* were diffusion activation energies in the *γ*/*α* interface, equal to 96,850 and 154,800 J·mol^−1^, for C and Cr, respectively. Equations (5) and (6) respectively expressed the C and Cr fluxes’ balance at the *γ*/*α* interface. In addition, Equations (7) and (8) respectively expressed the C and Cr fluxes’ balance at the *γ*/*θ* interface.

**Figure 3:**
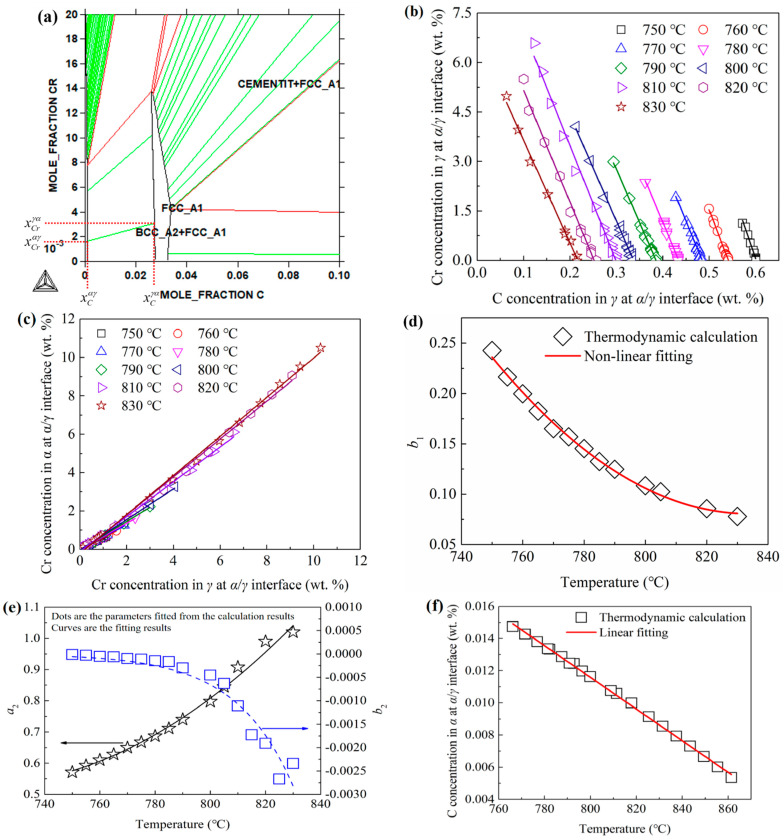
The thermodynamic equilibrium data for Fe-C-Cr system calculated with Thermo-Calc software. (**a**) Equilibrium phase diagram for ferrite and austenite at 750 °C; (**b**) relationships between the C and Cr concentrations in austenite at the *γ*/*α* interface at different temperatures; (**c**) the relationships between the Cr concentrations in ferrite and austenite at the *γ*/*α* interface at different temperatures; (**d**) relationship between *b*_1_ (in Equation (13)) and temperature; (**e**) relationships between *a*_2_ and *b*_2_ (in Equation (14)) and temperature; (**f**) relationship between the interfacial C concentration in ferrite and temperature.

If the local thermodynamic equilibriums at the phase interfaces are always satisfied during the austenitization process, the relationships between the interfacial solute concentrations can be fitted from the phase diagrams calculated with the Thermo-Calc software. Figure 3a shows the calculation results for the equilibrium phase diagram for ferrite and austenite of the Fe-C-Cr system at 750 °C. The tie-lines between ferrite and austenite showed the relationships between the interfacial concentration of C and Cr in the ferrite and austenite. As the boundary of ferrite was perpendicular to the abscissa, the interfacial C concentration in ferrite should be independent. Figure 3b shows the relationships between the C and Cr concentration in austenite at the interface between ferrite and austenite for different temperatures. Figure 3c shows the relationships between the Cr concentration in ferrite and austenite at the interface between ferrite and austenite for different temperatures. As shown in Figure 3b,c, all these relationships between the interfacial concentrations can be expressed with the following linear formulas:

(13) $$c_{Cr}^{\gamma \alpha }=a_{1}c_{C}^{\gamma \alpha }+b_{1},$$

(14) $$c_{Cr}^{\alpha \gamma }=a_{2}c_{Cr}^{\gamma \alpha }+b_{2},$$

As shown in Figure 3b, the slopes of the fitted lines were very close to each other; thus, the average value of the slopes for different temperatures was used as the value of *a*_1_. Figure 3d,f shows the non-linear relationships between the values of the linear fitted parameters (*b*_1_, *a*_2_ and *b*_2_) and temperature. These relationships were all expressed with comfortable non-linear formulas, which are shown in Table 1. Figure 3f shows the relationship between the interfacial C concentration in ferrite at the *α*/*γ* interface and temperature. The linear fitting results are also shown in Table 1.

Similar to the above calculation for the interfacial C and Cr concentrations at the *γ*/*α* interface, for the interface between austenite and cementite, the linear relationships between the interfacial Cr concentration and C concentration were also obtained. These relationships can be expressed with the following linear formulas:

(15) $$c_{Cr}^{\gamma \theta }=c_{1}c_{C}^{\gamma \theta }+d_{1},$$

(16) $$c_{Cr}^{\theta \gamma }=c_{2}c_{C}^{\gamma \theta }+d_{2},$$

Non-linear fitting for the relationships between the parameters (*c*_1_, *d*_1_, *c*_2_, *d*_2_) and temperature are also shown in Table 1. The C concentration in the cementite was calculated to be 0.067 wt.% for the present steel and changed little.

According to the results of the thermodynamic equilibrium calculation, the interfacial concentrations of C and Cr in Equations (5)−(8) can be expressed with *c_C_^γα^*, *c_C_^γθ^* and temperature. Thus, there were only four independent variables (*c_C_^γα^*, *c_C_^γθ^*, *v*_0_ and *S_θ_*) in Equations (5)–(8). The moving velocity of the pearlite/austenite interface was calculated by solving Equations (5)–(8) with the Newton iterative method.

When the temperature was above PNTT, the partition of the substitutional alloying element was not needed; the austenitization process was only controlled by the diffusion of C. Under this situation, only Equations (5) and (7) were used to calculate the pearlite/austenite interface moving velocity. The interfacial C concentrations in austenite can be calculated from the determined interfacial Cr concentrations with Equations (13) and (15). Thus, there are only two independent variables (*v*_0_ and *S_θ_*) in Equations (5) and (7). The interface moving velocity was calculated by solving Equations (5) and (7).

3. Cellular evolution rules

**Figure 4:**
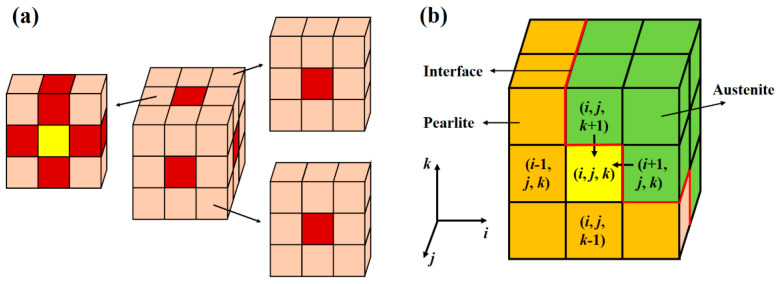
Schematic diagram for the 3D cellular automata cells. (**a**) The von-Neumann neighborhoods; (**b**) the evolution of the cells for the pearlite to austenite transformation process.

In the 3D cellular automata model, the pearlite microstructure was divided into small cubic cells. Every cell was represented with three feature parameters, including position coordinates (*i*, *j*, *k*); orientation vector (*a*, *b*, *c*); and austenite volume fraction *f_γ_* (when this value less than 1, the microstructure is pearlite; when this value is greater than or equal to 1, the microstructure is austenite). The evolution of a cell was decided by its neighbor cells. As shown in Figure 4a, the von-Neumann neighbors (containing the 6 cells’ surfaces contacting the center cell) were used to calculate the evolution of the center cell. As shown in Figure 4b, the transformed volume fraction of austenite for a pearlite cell (*i*, *j*, *k*) at the pearlite/austenite interface during Δ*t* (Δ*f_γ_* (*i*, *j*, *k*, Δ*t*)) could be calculated with the following formula:

(17) $$\begin{array}{l} \Delta f_{\gamma }(i,j,k,\Delta t)=\delta (i+1,j,k)v(i+1,j,k)\Delta t+\delta (i-1,j,k)v(i-1,j,k)\Delta t\\ +\delta (i,j+1,k)v(i,j+1,k)\Delta t+\delta (i,j-1,k)v(i,j-1,k)\Delta t\\ +\delta (i,j,k+1)v(i,j,k+1)\Delta t+\delta (i,j,k-1)v(i,j,k-1)\Delta t \end{array}$$

where Δ*t* was the time step; *v* (*i’*, *j’*, *k’*) was the moving velocity of the interface between the center cell (*i*, *j*, *k*) and its neighbor cell (*i’*, *j’*, *k’*). *δ* (*i’*, *j’*, *k’*) was a function: if the neighbor cell (*i’*, *j’*, *k’*) was austenite, the value of this function equaled 1; if the neighbor cell (*i’*, *j’*, *k’*) was pearlite, the value of this function equaled to 0. After *n* steps of iteration, the total volume fraction of austenite for an interfacial cell was:

(18) $$f_{\gamma }=\sum_{h=1,2,3,\ldots }^{n}\Delta f_{\gamma }^{h}(i,j,k,\Delta t),$$

When *f_γ_* ≥ 1, the interfacial cell changes to austenite, and the over transformed volume fraction of this cell (equal to *f_γ_*—1) will be equally distributed to its neighbor pearlite cells.

## 3. Results and Discussion

### 3.1. Experimental Results

The previous proposed model was used to calculate the transformation volume fraction of austenite based on dilatometric data [31]. The average atomic volume (*V*) of the samples could be expressed with the following formula:

(19) $$V=f_{\alpha }\cdot V_{\alpha }+f_{\theta }\cdot V_{\theta }+f_{\gamma }\cdot V_{\gamma }=(V_{\alpha }-V_{\gamma })\cdot f_{\alpha }+(V_{\theta }-V_{\gamma })\cdot f_{\theta }+V_{\gamma },$$

(20) $$f_{\theta }=A\cdot f_{\alpha }+B,$$

where *f_α_* and *f_θ_* were the volume fractions of ferrite and cementite, respectively. *V_α_*, *V_θ_* and *V_γ_* were the average atomic volumes of ferrite, cementite and austenite, respectively. *A* and *B* were two parameters related to the solute concentrations and densities of the phases [31]. The average atomic volumes of the phases can be described with the following formula according to their crystal structures:

(21) $$V_{\gamma }=\frac{1}{4}a_{\gamma }{}^{3},$$

(22) $$V_{\alpha }=\frac{1}{2}a_{\alpha }{}^{3},$$

(23) $$V_{\theta }=\frac{1}{12}a_{\theta }b_{\theta }c_{\theta },$$

where *a_γ_* and *a_α_* were the lattice constants of austenite and ferrite, respectively; *a_θ_*, *b_θ_* and *c_θ_* were the lattice constants of cementite. The lattice constants can be expressed with the following formulas:

(24) $$a_{\gamma }=a_{\gamma_{o}}[1+\beta_{\gamma }(T-300)],$$

(25) $$a_{\alpha }=a_{\alpha_{o}}[1+\beta_{\alpha }(T-300)],$$

(26) $$a_{\theta }=a_{\theta_{o}}[1+\beta_{\theta }(T-300)],$$

(27) $$b_{\theta }=b_{\theta_{o}}[1+\beta_{\theta }(T-300)],$$

(28) $$c_{\theta }=c_{\theta_{o}}[1+\beta_{\theta }(T-300)],$$

where *a_αo_* was the lattice parameter of ferrite at room temperature, taken to be that of pure iron (*a_α_*_o_ = 2.866 Å); *a_θo_*, *b_θo_* and *c_θo_* were the lattice parameters of cementite at room temperature [32], given by 4.5246, 5.0885 and 6.7423 Å, respectively; and *a_γo_* was the lattice parameter of austenite at room temperature as a function of the chemical composition of the austenite [33,34]:

(29) $$a_{\gamma_{o}}=3.573+0.033C+0.00095\mathrm{Mn}-0.0002\mathrm{Ni}+0.0006\mathrm{Cr}+0.0031\mathrm{Mo}+0.0018V,$$

where the chemical composition was measured in wt.%, and *a_γo_* was in Å.

*β_α_*_, *θ*, *γ*_ were the linear thermal expansion coefficients of ferrite, cementite and austenite, respectively, in K^−1^. The values of the linear thermal expansions of ferrite and austenite considered in these calculations were *β_α_* = 1.244 × 10^−5^ K^−1^ and *β_γ_* = 2.065 × 10^−5^ K^−1^. The thermal expansion coefficient of cementite increased with the temperature [32]. Using data published by Stuart and Ridley [32], the expression of the linear expansion coefficient as a function of temperature was:

(30) $$\beta_{\theta }=6.0\times 10^{-6}+3.0\times 10^{-9}(T-273)+1.0\times 10^{-11}{\left(T-273\right)}^{2},$$

where *T* was the temperature in K; *β_θ_* had the units of K^−1^.

Comparing the length of samples measured by dilatometer to the average atomic volume, the following relationship can be obtained:

(31) $$\frac{L^{3}}{L_{0}^{3}}=\frac{V}{V_{0}},$$

where *L* was the length of samples. *L*_0_ and *V*_0_ were the length of samples and average atomic volume at room temperature, respectively. Combining Equations (19), (20) and (31), the volume fraction of austenite can be calculated.

**Figure 5:**
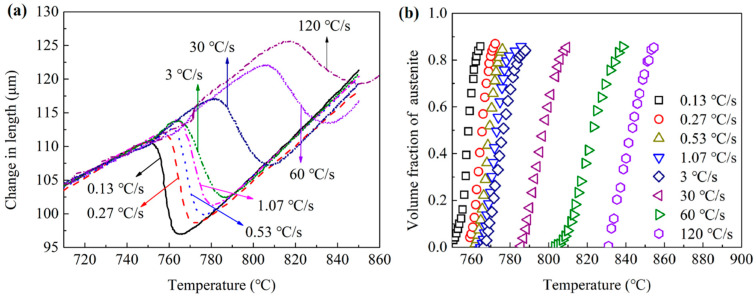
(**a**) The expansion curves for different heating rates; (**b**) the relationships between austenite volume fraction and temperature.

Figure 5a shows the dilatometric curves of the samples for heating rates of 0.13, 0.27, 0.53, 1.07, 3, 30, 60 and 120 °C/s. The average values of three repeated experiments were used. Part of the experimental data for one of the three repeated experiments can be found in [2,17]. The average coefficient of variation was 0.088. The curves showed an obvious decrease during the austenitization process, as the average atomic volume of austenite is smaller than pearlite. The *Ac*_1_ and *Ac*_3_ temperatures increased obviously with the heating rate. This should be owing to the time needed for the incubation of austenite nuclei that would improve the *Ac*_1_ temperatures for a higher heating rate. The deviations from linear expansion during heating before *Ac*_1_ were caused by the switching of the heating rate and the magnetic transition [35]. Figure 5b shows the volume fraction of austenite calculated from the dilatometric curves. The austenitization temperature for different heating rates changed from about 755 to 855 °C. This large temperature range shows the significant impacts of the heating rates on the austenitization processes. All the relationships between the volume fractions and temperatures were the typical sigmoid phase transformation curves, which corresponded to the nucleation and growth model. As the final austenite volume fractions were all lower than 1, the pearlite could not completely transform to austenite during the dissolution process of ferrite. The remaining cementite would be gradually dissolved into the austenite after the dissolution of ferrite during a much longer time.

**Figure 6:**
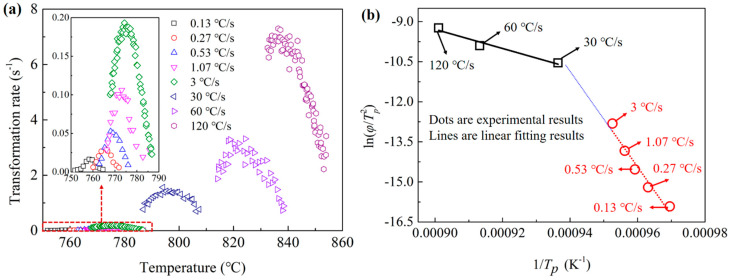
(**a**) The relationships between the transformation rate and temperature; (**b**) the relationships between ln(*φ*/*T_p_*^2^) and 1/*T_p_*.

The transformation activity energy of the diffusion-controlled phase transformation was always used to calculate with the Kissinger-like relationship:

(32) $$\ln\frac{\varphi }{T_{p}^{2}}=-\frac{E_{a}}{RT_{p}}+F,$$

where *E_a_* was the activity energy; *φ* was the heating rate; *T_p_* was the temperature at which the peak austenitic transformation rate was achieved; and *F* was a fitting parameter. Figure 6a shows the relationships between the pearlite-austenite transformation rate and temperature for different heating rates. The transformation rate obviously increased with the heating rate for the increasing in the transformation temperature. All the curves of transformation rates increased first and then decreased, showing the peak transformation rates at specific temperatures. According to Equation (32), there should be a linear relationship between ln(*φ*/*T_p_*^2^) and 1/*T_p_*, and the value of *E_a_* can be linearly fitted. Figure 6b shows the experimental results of ln(*φ*/*T_p_*^2^) and 1/*T_p_*. It can be easily found that the slopes were very different for low and high heating rates. However, the linear relationships between ln(*φ*/*T_p_*^2^) and 1/*T_p_* can be observed for both the low and high heating rates. The critical heating rate between the low and high heating rates was approximately 30 °C/s. The absolute value of the slope for heating rates under 30 °C/s was much larger than the value above 30 °C/s. The fitted values of the activity energies for low and high heating rates were 1.49 × 10^6^ and 2.98 × 10^5^ J·mol^−1^, respectively. 

### 3.2. DICTRA Simulation Results

The different activity energies mean that there should be different transformation mechanisms for low and high heating rates. The pearlite to austenite transformation process of the present steel can be controlled by two kinds of mechanisms: under the PNTT, the transformation was controlled by the diffusion of both C and Cr; and above the PNTT, the transformation was controlled only by the diffusion of C. For the first mechanism, the partition of Cr was required to maintain the carbon activity difference needed for the phase transformation. However, for the second mechanism, the partition of Cr was not needed, as the carbon activity difference can be always satisfied. 

**Figure 7:**
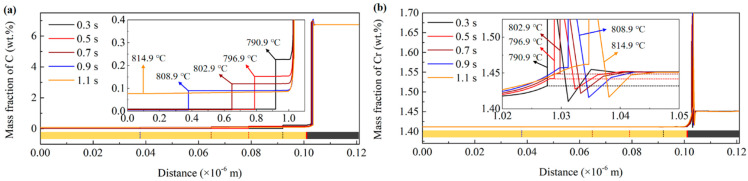
Temporal evolutions of the concentration distributions of (**a**) C and (**b**) Cr simulated with the DICTRA software.

To obtain the PNTT of the present steel, the DICTRA simulation of the austenitization process for the heating rate of 30 °C/s was implemented. Figure 7a,b shows the simulated C and Cr concentration during the austenitization process. As shown in Figure 7a, the concentration of C shows clear interfaces of ferrite, austenite and cementite. During the austenitization process, the austenite grew to the ferrite with a much faster velocity than to the cementite. This was due to the much higher C concentration in the cementite than in austenite and ferrite. Figure 7b shows that the concentration of Cr in austenite at the *γ*/*θ* interface increased with the temperature. When the temperature was 803 °C, the Cr concentration in austenite at the *γ*/*θ* was very close to the Cr concentration in the cementite. This means that the partition of Cr was not required for the diffusion of cementite at the temperature of about 803 °C. As shown in Figure 6a, when the heating rate was 30 °C/s, part of the pearlite transformed under 803 °C, and the rest transformed above 803 °C. The DICTRA simulation results also prove that the PLE to NPLE transition occurred at the heating rate of 30 °C/s. As shown in Figure 6b, the point for 30 °C/s was located at the fitted lines for both low and high heating rates. Thus, the true activity energy for 30 °C/s was difficult to obtain with the method of Kissinger.

### 3.3. Cellular Automata Simulation Results

#### 3.3.1. Comparisons between the Simulation Results and Experimental Results

**Figure 8:**
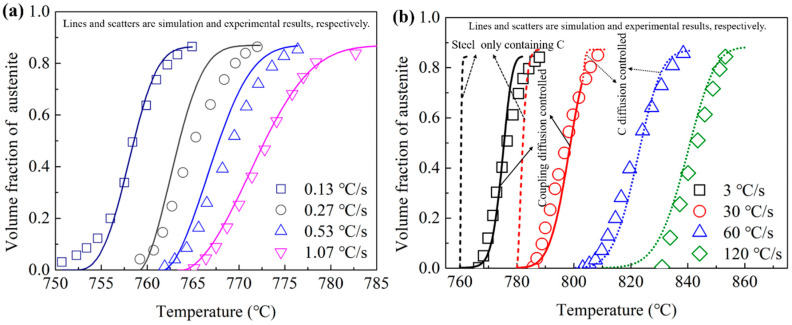
Comparisons of the austenitization kinetics between the experimental and cellular automata simulation results for the heating rate of (**a**) 0.13, 0.27, 0.53 and 1.07 °C/s; (**b**) 3, 30, 60 and 120 °C/s.

Figure 8 shows the comparisons between the simulation results and experimental results. As shown in Figure 8a,b, the lines and scatters show the simulation and experimental results, respectively. The solid lines show the simulation results for the temperature under the PNTT. Under this condition, the transformations were controlled by the coupling diffusion of C and Cr, as shown in Equations (5)–(8). The figure shows good agreements between the experimental results and the simulation results for coupling diffusion when the temperature was lower than the PNTT. As shown in Figure 8b, the dotted lines show the simulation results for the temperature above the PNTT. Under this condition, the transformations were controlled by the diffusion of C. The simulation results for 30 °C/s show the obvious increasing of the transformation rate when the temperature exceeded the PNTT. For the heating rate of 60 and 120 °C/s, the transformation temperature range was totally above the PNTT; thus, the transformation process was completely controlled by the diffusion of C. The simulation results and the experimental results also show good agreement with each other when the temperature exceeded the PNTT. 

As shown in Figure 8b, the dashed lines show the simulation results for a steel only containing C, in which the influence of Cr on the interfacial concentration of C was completely cancelled. As the transformation rates of the simulation results for the steel only containing C were much larger than the experimental results, the inhibiting effects of Cr on the austenitization transformation are very pronounced. 

In summary, the present proposed cellular automata model shows good predictions for the austenitization transformation processes of this Fe-C-Cr steel. As the Cr concentration difference of the initial ferrite and cementite of the present steel is low, the errors caused by the linear distribution assumption for the solute concentrations are weakened. 

#### 3.3.2. Influences of Pearlite Lamellae Orientation 

**Figure 9:**
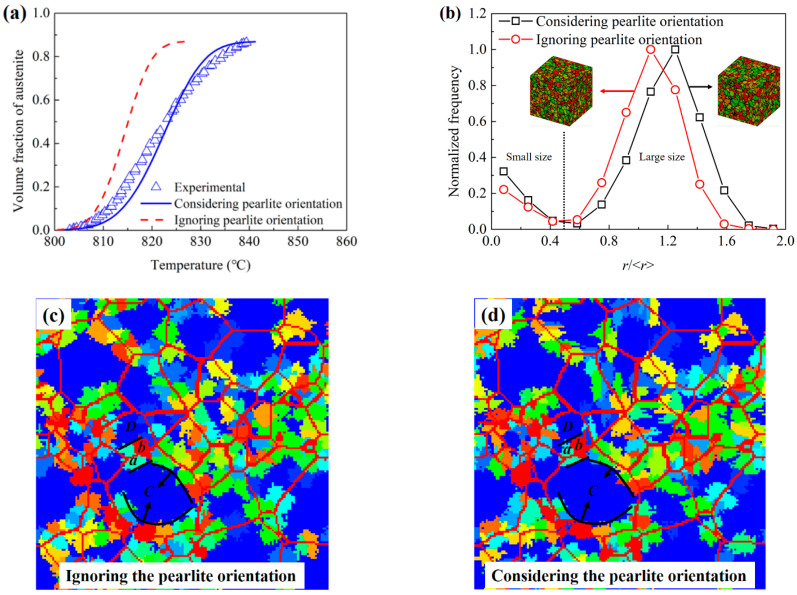
Simulation results for the models considering and ignoring the pearlite orientation. (**a**) Relationships between the austenite volume fraction and temperature; (**b**) grain size distribution; cross sections of the simulated microstructures for the models ignoring (**c**) and considering (**d**) the pearlite lamellae orientation.

The influences of the pearlite lamellae orientation on the austenitization kinetics and austenite grain distributions were also researched with the cellular automata simulation. Figure 9a shows the simulation results of the models considering and ignoring the pearlite lamellae orientations and the experimental results for the heating rate of 60 °C/s. As shown in the figure, when ignoring the influences of pearlite lamellae orientation, the transformation rate obviously improved and the finishing temperature of austenitization decreased from about 840 °C to 820 °C. Figure 9b shows the simulation results of the grain size distributions of the models considering and ignoring the pearlite lamellae orientation, where *r* and <*r*> were the radius of the grains and the average radius of all grains, respectively. The simulation results of the two models show the abnormally large numbers of small size grains. This should be due to the lack of grain coarsening during the rapid heating process. It also shows the larger frequency of small grains for the model considering the pearlite orientation than the model ignoring the pearlite orientation. This means that some small grains are inhibited to grow up for the small-included angle between the interfacial moving directions and the pearlite lamellae orientations. As shown in Figure 9b, in the large grain size region, the peak frequencies occurred at about *r*/<*r*> = 1.3 and 1.1 for the models considering and ignoring the pearlite lamellae orientation, respectively. The larger grain size for the model considering the pearlite lamellae orientation should be induced because some of the growth directions of the austenite grains were inhibited heavily, and the other grains growing in slightly inhibited directions would grow to larger sizes. Figure 9c,d shows the cross sections of the simulated microstructures for the models ignoring and considering the pearlite lamellae orientation when 20% of the pearlite transformed to austenite. The two figures have the same initial austenite nuclei distributed at the grain boundary. The blue matrix and the red net indicate the untransformed pearlite and the interfaces between the initial pearlite colonies, respectively. The small grains of other colors are the austenite grains transformed from the pearlite. As shown in Figure 9c,d, the two small austenite grains *a* and *b*, which nucleated at the interface between the two pearlite colonies *C* and *D*, both grew into their neighbor pearlite colonies. Comparing the grain boundary moving distances of grain *a* and *b* into the pearlite colonies *C* and *D*, the simulation results of the model considering the pearlite lamellae orientation show the obviously different grain boundary moving distances into colonies *C* and *D* for the different pearlite lamellae orientation of the colonies *C* and *D*. However, for the simulation results of the model ignoring the pearlite lamellae orientation, the grain boundary moving distances into colonies *C* and *D* were almost the same. Thus, the austenite grains tend to grow into their easy growing neighbor pearlite colonies. As the orientations of their neighbor pearlite colonies are randomly distributed, some austenite grains will have more opportunities to grow up, but some other austenite grains will heavily inhibited. This should be the reason why the simulation results of the model considering the pearlite orientation had more small size grains, as shown in Figure 9b. Comparing the austenite grains nucleated at different directions of the boundary of the colony *C*, as shown in Figure 9d, the austenite grains nucleated at the lower left of the colony boundary grew more easily into colony *C* than the grains nucleated at the upper right of the colony boundary. However, for the simulation results of the model ignoring the pearlite orientation, the austenite grains nucleated at a different direction of the colony boundary almost had the same growth velocity. Thus, due to the influence of the pearlite lamellae orientation, the sizes of the austenite grains nucleated at the easy growing direction of a pearlite colony were larger than other grains. This should be the reason why the simulation results of the model considering the pearlite orientation had larger grains, as shown in Figure 9b.

#### 3.3.3. Comparison between the Rapidly Austenitized Grains and Normal Grains

**Figure 10:**
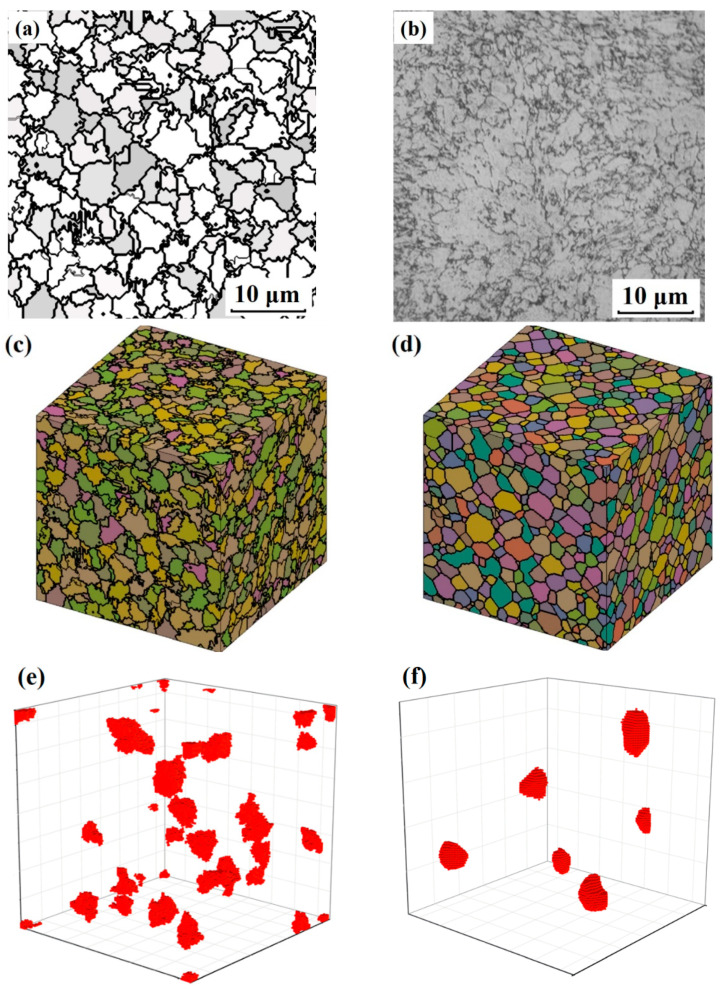
(**a**) Cross section of the simulated microstructure for the heating rate of 30 °C/s; (**b**) optical micrograph of the austenite microstructure after rapid heating and cooling; (**c**) simulation results of the 3D microstructure for 30 °C/s; (**d**) 3D normal grain microstructure simulated with classical Monte Carlo model; (**e**) 3D single grains drawn from (**c**); (**f**) 3D single grains drawn from (**d**).

Figure 10a,b shows the simulation and experimental results of the austenite microstructures after being austenitized with the heating rate of 30 °C/s. The simulation and experimental results both show irregularly shaped grains. This should be related to the random nucleation locations of austenite and their various growing rates in different directions. As the samples were rapidly heated and cooled, the grains had little time to coarsen (which would lead to the homogenization of grains by grain boundary moving and swallowing of small grains). Figure 10c,d shows the 3D microstructures simulated with the present cellular automata model for the heating rate of 30 °C/s, and the normal 3D grains simulated with a classical Monte Carlo grain coarsening model, respectively. Figure 10e,f shows the single grains drawn from the 3D microstructures of Figure 10c,d, respectively. Comparing the grains in Figure 9e,f, it can be observed that the rapidly austenitized grains had more rough surfaces, sharp crystal angles and irregular shapes than normal austenite grains. 

**Figure 11:**
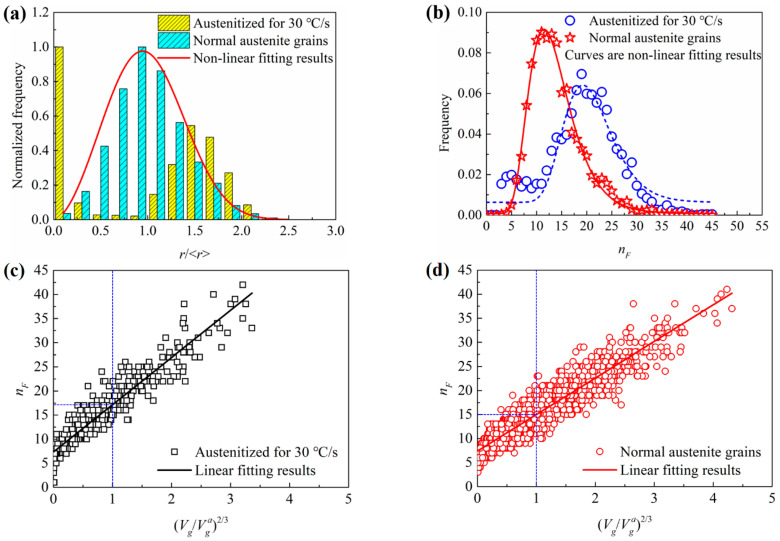
(**a**) Grain size distributions and (**b**) grain face number distributions of the grains austenitized for 30 °C/s and normal grains. Relationships between the grain face number and (*V_g_*/*V_g_^a^*)^2/3^ of (**c**) the grains austenitized for 30 °C/s; and (**d**) the normal grains.

Figure 11a shows the grain size distributions of the microstructures austenitized for 30 °C/s and the normal grains. As shown in the figure, the size distribution of the normal grains could be reasonably non-linear fitted with the following Weibull distribution function:

(33) $$f(u)=\frac{\xi }{\omega^{\xi }}u^{\xi -1}\exp\left(-{\left(\frac{u}{\omega }\right)}^{\xi }\right),\;u=r/<r>,$$

where *ω* and *ξ* were two fitting parameters. As shown in Figure 11a, for the normal grains, the critical grain radius (the radius of maximum frequency) was very close to the average grain radius. However, the grain size distribution of the rapidly austenitized microstructure seriously deviated from the normal grain size distribution. Two frequency peaks in the small and large grain size regions were observed in the rapidly austenitized microstructure. As the coarsening time of the rapidly austenitized microstructure was very short, much more grains with a small radius were observed. In the large grain size region, another frequency peak was located at *r*/<*r*> = 1.5. The abnormal large frequency distribution of small grains led to the decrease in the average radius, which induced the deviation of the critical grain radius to the larger side. Figure 11b shows the distributions of grain face numbers, which were obtained by counting the number of grains’ surfaces contacting with a grain. As shown in the figure, the grain face number distributions of the microstructures austenitized for 30 °C/s and the normal grains were non-linearly fitted with the following log-normal distribution functions:

(34) $$g(n_{F})=\frac{1}{\sqrt{2\pi }\sigma n_{F}}\exp\left(-\frac{{\left(\ln n_{F}-\mu \right)}^{2}}{2\sigma^{2}}\right),$$

where *n_F_* was the face number of the grains; *σ* and *μ* were the two fitting parameters. The critical face number (the face number of maximum frequency) of the 3D normal grains was about 12. However, the critical grain face number of the rapidly austenitized microstructure showed a much larger value of about 20, which means much a larger grain boundary area and more irregular grain shapes. The figure also shows a small frequency peak in the small grain face number region at about five faces. This should be corresponding to the small grains which always had fewer neighbor grains. The average face number of the rapidly austenitized microstructure was 18.9, which was also much larger than the average normal grain face number of 14.1. 

According to the research of Ding et al. [36], the relationship between face number and relative volume of grains could be expressed with the following formula:

(35) $$n_{F}=\lambda {\left(V_{g}/V_{g}^{a}\right)}^{2/3}-\eta ,$$

where *V_g_* and *V_g_^a^* were the volume of a grain and the average volume of all grains, and the expression $V_{g}/V_{g}^{a}$ was the relative grain volume; *λ* and *η* were two fitting parameters. As shown in Figure 11c,d, the grains rapidly austenitized for 30 °C/s and normal austenite grains both showed obvious linear relationships between the grain face number and ${\left(V_{g}/V_{g}^{a}\right)}^{2/3}$. The linear fitting results show very close intercepts of Equation (35) for the two kinds of grains: 7.3 for the rapidly austenitized grains and 7.4 for the normal grains. This means that the average grain face numbers of the smallest grains for the two kinds of grains were very close to each other. Thus, the influence of the microstructure evolution on the face number of the vanishing grains was little. Comparing Figure 11c,d, the maximum grain face number of the two kinds of grains were also very close to each other, which means that the influence of microstructure evolution on the maximum grain face number was also little. However, the linear fitting results show that the slopes of Equation (35) for the two kinds of grains, which were 9.8 for rapidly austenitized grains and 7.6 for normal grains, were very different from each other. This means that more grain faces are needed to form the rapidly austenitized grains than the normal austenite grains with the same relative volume. The figures also show that the average grain face numbers of the grains had an average grain volume for the rapidly austenitized grains and normal austenite grains of 17.4 and 15, respectively. This also means there was a redundancy of the grain faces for rapidly austenitized grains when comparing to the normal austenite grains.

According to the studies on grain scale interactions [37,38,39], the different grain shapes and distributions will lead to different mechanical properties. Thus, the topological and size distribution differences between the rapidly austenitized and normal grains should be important resources of their distinguished mechanical properties.

## 4. Conclusions

To predict the pearlite to austenite transformation of the Fe-C-Cr low alloy steel, a novel 3D cellular automate model that considered the influences of coupling diffusion of C and Cr, the interfacial diffusion between pearlite lamellae and the pearlite lamellar orientations was established basing on the thermodynamic equilibrium data obtained from the Thermo-Calc software, and the simulation results of DICTRA module. The austenitization kinetics of the GCr15 bearing steel during uniform heating processes were experimentally researched to clarify the influence of the heating rate and validate the newly proposed model. According to the experimental and simulation results, the following conclusions were obtained:

(a) The good agreements between the experimental results and the simulation results of the present cellular automata model indicate that the newly proposed model is reasonable.
(b) The experimental results show an obvious change of the transition activity energy from low to high heating rates, which means a change of the transformation mechanisms. The activity energies of the austenitization processes were obtained to be 1.49 × 10^6^ J·mol^−1^ and 2.98 × 10^5^ J·mol^−1^ for the heating rate under and above 30 °C/s, respectively. The influences of the heating rate on the transformation mechanism could be clearly observed.
(c) The DICTRA simulation results show that the austenitization transformation is controlled by the couple diffusion of C and Cr below the PNTT and by the diffusion of C above the PNTT. The PNTT of the present steel is calculated to be 803 °C. According to the experimental results, this temperature was located in the temperature range for the heating rate of 30 °C/s. The DICTRA simulation proved that the experimental observed mechanism transition was the partition to non-partition transition.
(d) The lamellar orientation influences both the austenitization kinetics and the topological aspects of the austenite grains. Therefore, considering the influences of the lamellar orientation in an austenitization model is necessary.
(e) The topological aspects of the austenite grains obtained via rapid heat treatment were very different from the normal grains. The rapidly austenitized grains have more rough surfaces, sharp crystal angles and irregular shapes than normal austenite grains.

## Figures and Tables

**Table 1:** The parameters for the thermodynamic equilibrium relationships between the solute concentrations of Fe-C-Cr system.

*α*/*γ* Interface	*θ*/*γ* Interface
$a_{1}=-35.36$	$c_{1}=-7.71\times 10^{-5}T^{2}+0.11T-44.77$
$b_{1}=2.21\times 10^{-5}T^{2}-0.037T+15.46$	$d_{1}=2.09\times 10^{-6}T^{2}-0.0031T+1.21$
$a_{2}=3.91\times 10^{-5}T^{2}-0.056T+20.52$	$c_{2}=-3.39\times 10^{-4}T^{2}+0.62T-339.82$
$b_{2}=9.48\times 10^{-21}\times (1-1.05^{T})$	$d_{2}=5.01\times 10^{-6}T^{2}-0.0074T+3.28$
$c_{C}^{\alpha \gamma }=-9.86\times 10^{-7}T+9.03\times 10^{-4}$	$c_{C}^{\theta \gamma }=0.067$

## Data Availability

Not applicable.
